# Delivery Route Following Elective Induction of Labor at Term: Analysis of 807 Patients

**DOI:** 10.4021/jocmr1476w

**Published:** 2013-06-21

**Authors:** Meghan I. Rattigan, Andrew L. Atkinson, Jonathan D. Baum

**Affiliations:** a Department of Obstetrics and Gynecology, Jersey Shore University Medical Center, Neptune NJ, USA

**Keywords:** Elective induction, Operative delivery, Cesarean section

## Abstract

**Background:**

The purpose of this study is to compare mode of delivery for both nulliparous and multiparous women at term that underwent elective induction of labor to those who arrived in spontaneous labor.

**Methods:**

Medical records of 807 deliveries were reviewed. There were 566 labor patients and 241 elective induction patients.

**Results:**

Women who underwent elective induction of labor were more likely to undergo cesarean delivery compared to those women who arrived in spontaneous labor (41.1% versus 9.9%, P = 0.001). This was true for both nulliparous women (49% versus 31%, P < 0.0001), and multiparous women (22.7% versus 1.6%, P < 0.0001). The rate of operative vaginal delivery was also increased in the elective induction of labor group (8.4% versus 3.6%, P < 0.0001). Operative vaginal delivery was statistically significant in multiparous women (21% versus 4.1%, P < 0.0001), but not in nulliparous women (10.1% versus 9.8%, P = NS).

**Conclusion:**

Elective induction of labor at term is associated with an increased risk of cesarean section in both nulliparous and multiparous women. There is also an increased risk of an operative vaginal delivery in multiparous women who underwent elective induction of labor.

## Introduction

Approximately 1 in 4 women in the United States are induced; with up to 1 in 10 being induced for elective reasons [1]. Elective induction of labor is defined as initiation of a term labor without medical or obstetric indications [2]. Induction of labor has long been associated with increased risk of cesarean section especially in nulliparous women with an unfavorable cervix. However, the outcomes of numerous studies dealing with elective induction cannot be generalized for all women undergoing induction. Most studies include a wide spectrum of gestational age with multiple indications for induction, with a majority failing to separate medically indicated versus elective indications [3-5]. Medically indicated reasons for induction such as preeclampsia, premature rupture of membranes, severe fetal growth restriction, and oligohydramnios are independent risk factors for cesarean delivery, with an increased risk of 17.7% [6, 7].

The incidence of elective induction appears to be increasing at a greater rate than medically indicated inductions and now make up over one third of the total delivery population [8-10]. In 1990, the rate of induction was 9.5%, with a sharp increase to 23% of total deliveries in 2008-a relative increase of 143% [11].

The purpose of our study is to compare mode of delivery for both nulliparous and multiparous women at term that underwent elective induction of labor compared to those who arrived in spontaneous labor.

## Materials and Methods

We conducted a retrospective cohort study of nulliparous and multiparous women at term (between 37 and 41 weeks 6/7 days gestation) that underwent elective labor induction (induction group) and compared them to women in spontaneous labor (labor group) in 2011 at Jersey Shore University Medical Center, a community teaching hospital with a perinatal referral center. Institutional review board approval was obtained. The primary outcome for our study was cesarean delivery and the secondary outcome was operative vaginal delivery (either vacuum or forceps). Patients were excluded if they were not between 37 and 41 weeks 6/7 days gestational age, had a fetal demise, prior cesarean delivery, multiple gestation, or if there was maternal or fetal indication for delivery. Patients were also excluded if their pregnancy dates were not confirmed by a first trimester ultrasound.

Pearson’s Chi-squared test with Yates’ continuity correction was used to compare proportions between the two groups. The two groups did not differ in regard to maternal age, body mass index (BMI), and gestational age. Demographic and clinical data for both groups are shown in Table 1.

**Table 1:** Patient Demographics

Characteristics (Mean ± SD)	Induction (n = 241)	Labor (n = 566)	P Value
Patient Age	29.5 ± 6.1	28.0 ± 6.2	NS
Gestational Age (weeks)	39.9 ± 1.0	39.3 ± 1.0	NS
Body Mass Index (kg/m^2^)	30.1 ± 5.8	28.7 ± 5.4	NS
Nulliparous (n, %)	122 (51%)	247 (44%)	-
Multiparous (n, %)	119 (49%)	319 (56%)	-
Cervical dilation on admission (cm)	1.8 ± 1.0	4.5 ± 2.0	< 0.0001
Cervical effacement on admission (%)	50.3 ± 21.5	78.8 ± 18.0	< 0.0001
Fetal station on admission	-2.4 ± 0.8	-1 ± 1.0	< 0.0001

NS: Not significant.

## Results

**Figure 1:**
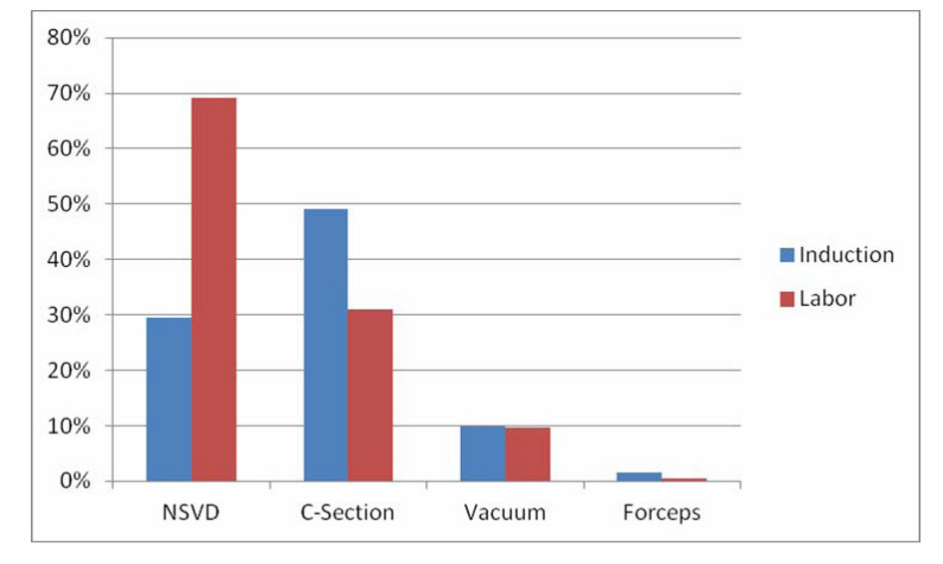
Outcomes for nulliparous patients.

**Figure 2:**
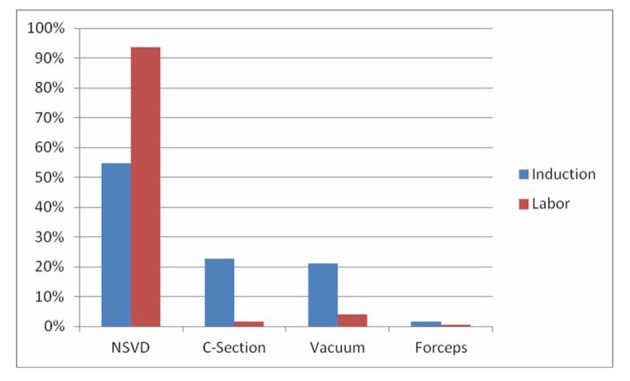
Outcomes for multiparous patients.

Women who underwent elective induction of labor were more likely to undergo cesarean delivery compared to those women who arrived in spontaneous labor (41.1% versus 9.9%, P = 0.001). This was true for both nulliparous women (49% versus 31%, P < 0.0001), and multiparous women (22.7% versus 1.6%, P < 0.0001). The rate of operative vaginal delivery was also increased in the elective induction of labor group (8.4% versus 3.6%, P < 0.0001). Operative vaginal delivery was statistically significant in multiparous women (21% versus 4.1%, P < 0.0001), but not in nulliparous women (10.1% versus 9.8%, P = NS). Among the 81 women delivered by operative vaginal delivery 74% were via vacuum and 7% were via forceps. Mode of delivery is shown for nulliparous and multlparous women in Figure 1, 2.

## Discussion

In 2008, the overall induction of labor rate in the United States was 23.1%, with roughly 1 in 5 pregnant women having their labor induced. Induction of labor has been identified as a contributing factor to the rising rate of cesarean deliveries in the United States [12].

In the present study, we examined outcomes of nulliparous and multiparous women undergoing elective induction of labor and compared these outcomes to women arriving in spontaneous labor. We found the rates of cesarean delivery were higher in both nulliparous and multiparous women who underwent elective induction of labor. We also found the rates of operative vaginal delivery were higher mulitparous women who underwent elective induction of labor.

The cesarean section rate in the United States in 2011 was 32.8%, with many individual institutions at or greater than 50% [13, 14]. While reduction of the current cesarean rate is a worthwhile goal, the concern of many is that elective induction of labor is thought to be a major driving force for this current trend. However, despite the efforts of several institutions and academic groups, no single reason for the increasing cesarean rate has been established.

In regard to patient preference and autonomy, Out et al [15] reported that up to 50% of women would choose elective induction based on psychological reasons and past obstetric complications. Knoche et al [16] reported that nearly 58% of women stated that “getting the pregnancy over with” was their motivation for the use of elective induction, while only 33% wanted to avoid medical induction. We would hope that no prudent practitioner or patient would go ahead with elective induction of labor with an unfavorable cervix unless sufficient time is to be allotted for cervical ripening.

To our knowledge, there has been no randomized study comparing mode of delivery in women undergoing elective induction of labor to women in spontaneous labor. In addition, the definition of failed induction remains undefined. Neither Gabbe Obstetrics, Williams’ Obstretric, nor the American College of Obstetricians and Gynecologists (ACOG), has defined failed induction [12, 17, 18]. Rouse et al [19], proposed a criteria for failed induction in 2000. In 2011, the Eunice Kennedy Shriver National Institute of Child Health and Human Development (NICHD) and Maternal-Fetal Medicine Units Network (MFMU) reported making failed induction an objective diagnosis, but they too acknowledge that causation could not be established due to labor management that was not standardized [20]. At the time of this writing, no standard definition for failed induction has been adopted. Despite several attempts at standardization, labor management has significant variation. We too caution the readers of this study and other studies that examine elective induction to avoid confusing association and cause. While elective induction of labor appears to increase rates of cesarean delivery, the exact mechanism to account for this increase remains unclear. A randomized trial is needed to confirm causation.

In 1998, Prysak et al [21], and in 2011, Osmundson [5] reported on elective induction versus spontaneous labor at term. Neither group found a significant difference in operative vaginal delivery rates. We may be the first to report that elective induction of labor increases the rate of operative vaginal delivery in the multiparous patient.

### Conclusion

We have shown in this retrospective cohort study that women undergoing elective induction of labor have increased rates of cesarean delivery, and that multiparous women have increased rates of operative vaginal delivery. Criteria for failed induction of labor and standardization of labor management are needed and warrant further study.
